# Smartphone addiction, anxiety, depression and stress in Mexican nursing students

**DOI:** 10.15649/cuidarte.3814

**Published:** 2024-09-01

**Authors:** Cornelio Bueno-Brito, Eduardo Pérez-Castro, Josefina Delgado-Delgado

**Affiliations:** 1 Facultad de Enfermería No.2, Universidad Autónoma de Guerrero, Acapulco, Guerrero, México. E-mail: buenobritoc@gmail.com Universidad Autónoma de Guerrero Facultad de Enfermería No.2 Universidad Autónoma de Guerrero Acapulco Guerrero Mexico buenobritoc@gmail.com; 2 Unidad de Investigación de Salud en el Trabajo, Instituto Mexicano del Seguro Social, Ciudad de México, México. E-mail: percasedu@gmail.com Instituto Mexicano del Seguro Social Unidad de Investigación de Salud en el Trabajo Instituto Mexicano del Seguro Social Ciudad de México Mexico percasedu@gmail.com; 3 Facultad de Enfermería No.2, Universidad Autónoma de Guerrero, Acapulco, Guerrero, México. E-mail: jose delgado001@yahoo.com.mx Universidad Autónoma de Guerrero Facultad de Enfermería No.2 Universidad Autónoma de Guerrero Acapulco Guerrero Mexico jose delgado001@yahoo.com.mx

**Keywords:** Smartphone Addiction, Students, Nursing, Depression, Anxiety, Emotional Stress, Adicción al Teléfono Móvil, Estudiantes de Enfermería, Depresión, Ansiedad, Estrés Emocional, Dependência de Smartphones, Estudantes de Enfermagem, Depressão, Ansiedade, Estresse Emocional

## Abstract

**Introduction:**

Cell phones have increased as a new communication technology in the modern world.

**Objective:**

To determine whether smartphone addiction is significantly associated with depression, anxiety, and stress among university nursing students in Acapulco, Guerrero, Mexico.

**Material and Methods:**

This descriptive and cross-sectional study involved 212 students who voluntarily participated. Data were collected using two questionnaires: the Smartphone Addiction Scale Short Version (SAS-SV) and the Depression Anxiety and Stress Scale (DASS-21). The information was then analyzed using descriptive statistics and linear and simple logistic regression models.

**Results:**

46.70% (99) use their phones for more than 5 hours a day, and 38.20% (68) of the students presented smartphone addiction. Simple linear regression models showed a significant association between SAS-SV scores and DASS- 21 subscale scores. Simple logistic regression models indicated that students with cell phone addiction are 2.57 times more likely to suffer from depression, 2.50 times more likely to experience anxiety, and 3.34 times more likely to suffer from stress compared to those without cell phone addiction.

**Discussion:**

Cell phone addiction was associated with such mental disorders among Mexican university students.

**Conclusions:**

These results could assist educational authorities in developing and implementing strategies to prevent depression, anxiety, and stress associated with smartphone use.

## Resumen.

**Introducción:**

El uso de los teléfonos móviles ha aumentado como nueva tecnología de comunicación en el mundo moderno.

**Objetivo:**

Determinar si la adicción a los teléfonos inteligentes se asocia significativamente con la depresión, la ansiedad y el estrés entre estudiantes universitarios de enfermería en Acapulco, Guerrero, México.

**Materiales y Métodos:**

Estudio descriptivo y transversal en 212 estudiantes que aceptaron voluntariamente participar en el estudio. Los datos se recopilaron mediante dos cuestionarios que incluyen la Escala de Adicción al Smartphone Versión Corta (SAS-SV) y la de Depresión, Ansiedad y Estrés (DASS-21). Posteriormente la información se analizó mediante estadísticas descriptivas, modelos de regresión lineal y logística simple.

**Resultados:**

El 46,70% (99) utiliza el teléfono más de 5 horas al día. El 38,20% (81) de los estudiantes presentó adicción a los teléfonos inteligentes. Los modelos de regresión lineal simple mostraron que las puntuaciones del SAS-SV se asociaron significativamente con las puntuaciones de las subescalas del DASS-21. Respecto a los modelos de regresión logística simple, se encontró que los estudiantes con adicción al teléfono móvil tienen 2,57 veces más probabilidad de padecer depresión, 2,50 de presentar ansiedad y 3,34 de sufrir estrés comparado con los estudiantes que no tienen adicción al teléfono móvil.

**Discusión:**

La adicción al teléfono móvil se asoció con dichos trastornos mentales en estudiantes universitarios mexicanos.

**Conclusiones:**

Los resultados del estudio podrían ayudar a las autoridades educativas a desarrollar e implementar estrategias para prevenir la depresión, la ansiedad y el estrés asociados con el uso de los teléfonos inteligentes.

## Resumo.

**Introdução:**

O uso de telemóveis tem aumentado como uma nova tecnologia de comunicação no mundo moderno.

**Objetivo:**

Determinar se a dependência do smartphone está significativamente associada à depressão, à ansiedade e ao stress entre estudantes universitários de enfermagem em Acapulco, Guerrero, México.

**Materiales e Métodos:**

Estudo descritivo e transversal em 212 estudantes que aceitaram voluntariamente participar no estudo. Os dados foram recolhidos através de dois questionários, incluindo a Smartphone Addiction Scale Short Version (SAS-SV) e a Depression, Anxiety and Stress Scale (DASS-21). Os dados foram depois analisados com recurso a estatísticas descritivas, regressão linear e modelos logísticos simples.

**Resultados:**

46,70% (99) utilizam o telemóvel mais de 5 horas por dia. 38,20% (81) dos estudantes revelaram dependência do telemóvel. Os modelos de regressão linear simples mostraram que as pontuações da SAS- SV estavam significativamente associadas às pontuações da subescala DASS-21. Em relação aos modelos de regressão logística simples, verificou-se que os estudantes com dependência de telemóvel tinham 2,50 vezes mais probabilidades de ter depressão, 2,57 vezes mais probabilidades de ter ansiedade e 3,34 vezes mais probabilidades de ter stress em comparação com os estudantes sem dependência de telemóvel.

**Discussão:**

A dependência do telemóvel foi associada a estas perturbações mentais em estudantes universitários mexicanos.

**Conclusões:**

Os resultados do estudo podem ajudar as autoridades educativas a desenvolver e implementar estratégias para prevenir a depressão, a ansiedade e o stress associados à utilização de smartphones.

## Introduction

A smartphone is a portable device that combines the traditional features of a cell phone with those of a handheld computer or touch tablet. Its name, "smart," comes from the fact that can perform many complex functions in addition to simply allowing two people to communicate over the phone^1^. Today, approximately 6.6 billion people worldwide use smartphones, and that number is expected to reach 7.8 billion by 20282. According to the results of the National Survey on the Availability and Use of Information Technologies in Households 2020 (ENDUTIH-2020) in Mexico, 88. ^2^ million people were cell phone users, of which 91.8% of cell phone users had a smartphone^3^.

Due to the increase in smartphone use in recent years, a growing number of studies have been conducted on smartphone addiction (SA) ^4^^, ^^9^. Some of these studies have reported a high prevalence of SA among young adults^4^^, ^^5^. A study conducted among dental students in Saudi Arabia^4^ found a prevalence of SA of 71.9%, while among medical students in India^5^, it was 85.4%. On the other hand, the prevalence of SA among students in a public university in Malaysia^6^ was 47.9%. However, other studies have reported a moderate prevalence of SA. A recent study of university students^7^^)^ found that 37.9% of students had SA, whereas in a cross-sectional study of 1441 university medical students in China, the prevalence of SA among participants was 29.8% (30.3% in men and 29.3% in women) ^8^.

Smartphone use is associated with numerous daily life dysfunctions that affect physical and mental health, social relationships, and academic and professional achievements^9^. Physical problems resulting from SA include neck and wrist pain, eye discomfort, and sleep problems, as well as depression, anxiety, and stress^9^^, ^^13^.

Regarding the relationship between common mental disorders and SA, a study conducted among medical students in Belgrade and Nis (central Serbia) ^14^ found that SA was associated with increased levels of stress (OR = 1.75, p = 0.003), anxiety (OR = 2.04, p < 0.001), and depression (OR = 2.29, p < 0.001). On the other hand, in a study conducted on a population of medical students in Changsha, China, SA was associated with depression, stress, and anxiety^15^. One study found that SA was positively correlated with depression (r = 0.375; p <0.01), anxiety (r = 0.253; p <0.01), and stress (r = 0.328; p <0.05) in Palestinian university students7. In this sense, a cross-sectional study of Tunisian students in public universities found that higher SA scores were significantly and positively correlated with scores on the depression (r = 0.474), anxiety (r = 0.499), and stress (r = 0.461) subscales^16^. On the other hand, research conducted among medical students in Iran^17^ found a significant relationship between excessive cell phone use and stress (p = 0.010) and anxiety (p = 0.028) using a multiple linear regression model; however, there was no significant relationship between the excessive cell phone use scale and depression (p = 0.075).

Most studies of SA have been conducted in Asian countries^4^^, ^^8^^, ^^15^^, ^^17^. To the best of our knowledge, there are no studies in Mexico that have examined the prevalence of SA among university students and whether there is an association with psychological disorders that affect mental health, mainly depression, anxiety, and stress. For these reasons, the objectives of this study are 1) to determine the prevalence of SA; 2) to determine the prevalence of depression, anxiety, and stress; and 3) to investigate the relationship between these mental disorders and SA among nursing students at a public university in southern Mexico.

## Materials and Methods

### Study design

A cross-sectional analytical research design was used in this study.

### Participants

This study was conducted during the months of September and October and involved 212 first- semester undergraduate students enrolled in the bachelor's degree program in nursing at the Nursing Faculty No. 2 of the Universidad Autónoma de Guerrero, the main public university in the state. The Nursing Faculty No. 2 has an enrollment of approximately 1,558 students, of which 414 were in their first year. First-year students were purposefully selected because they were more feasible to locate, as students in more advanced years were in clinical, social service, or professional practice. The sample was obtained by convenience and consisted of nursing students who met the following inclusion criteria: (1) being 18 years or older at the time of data collection, (2) owning and using a smartphone, and (3) having access to the Internet access via their phone. Before students decided to participate in the study, they were informed of the study's objectives, confidentiality and anonymity, and their right to refuse to participate without academic repercussions.

### Instruments

The survey was conducted in the Google Forms application and consisted of four sections to collect the information: 1) Sociodemographic data such as age, sex, marital status, and people with whom they lived at the time of the study; 2) questions related to cell phone use; 3) the Depression, Anxiety, and Stress Scale (DASS-21); and 4) the short version of the Smartphone Addiction Scale (SAS-SV). The survey link was emailed to students and was available for approximately two weeks to increase the response rate.

### Depression, Anxiety, and Stress Scale - DASS-21

The DASS-21 is a 21-item self-report measure with three subscales that measure the prevalence of anxiety, depression, and stress^18^. Each item is scored on a scale ranging from 0 (did not affect me at all) to 3 (affected me very much or most of the time). The cut-off points considered for depression are 5-6 mild, 7-10 moderate, 11-13 severe, and 14 or more, extremely severe. For anxiety, the cut-off points are 4-5 mild, 6-7 moderate, 8-9 severe, 10 or more, extremely severe. For stress, the cut-off points are: 8-9 mild, 10-12 moderate, 13-16 severe, and 17 or more extremely severe. The original scale study reported high reliability of the DASS-21; Cronbach's alpha (a) coefficients for depression, anxiety, and stress were 0.91, 0.84, and 0.90, respectively. Henry and Crawford^19^ found this scale to have good concurrent and discriminant validity and excellent internal consistency (a = 0.93).

### Smartphone Addiction Scale-SAS-SV

The SA was assessed with the SAS-SV, in its short version in Spanish, adapted by López-Fernández^20^, a scale developed and validated from the Smartphone Addiction Scale (SAS) proposed by Kwon in 2013^21^. It consists of 10 questions on a 6-point scale ranging from 1 (strongly disagree) to 6 (strongly agree). The minimum possible score is 10, and the maximum is 60. The SAS-SV cut-off point for diagnosing SA is different for men and women. For men, a score >31 indicates the presence of SA; for women, a score >33. The SAS-SV in Spanish, which we used in our study, has good psychometric properties and showed adequate internal reliability (a = 0.88) ^21^.

### Pilot Test

We conducted a pilot test of the questionnaire with 16 students. Participants evaluated the clarity, relevance, and pertinence of the questions. The estimated response time was between 8 and 12 minutes.

### Ethical considerations

Approval from the Ethics Committee was obtained by providing the following assurances: the study authors fully explained to the university students, through informed consent, their right to freely choose to participate, the study’s objectives, research methods, and protection of their privacy, confidentiality, and dignity. The data collected are used exclusively for research purposes.

The research is considered risk-free for the participants since it is a study in which an anonymous questionnaire is used, no direct intervention or intentional modification of variables is performed, and no experiments on humans or animals are performed during the process.

The research project adheres to both national and international standards for research involving human subjects. It complies with the principles outlined in the General Health Law and its regulations on Health Research (articles 13, 14, 16, and 17), as well as the Helsinki Declaration.

### Statistical analysis

The generated dataset was stored in Mendeley Data^22^. The median (interquartile range) was calculated for continuous variables. Frequencies (percentages) were calculated for categorical variables. The students were divided into two groups based on the total score obtained on the SAS- SV: SA and non-SA. The chi-squared, Fisher's exact, and Mann-Whitney U tests were used to compare differences between the two groups on the DASS-21 depression, anxiety, and stress subscales. To assess the linear relationship between the SAS-SV scores (independent variable) and the scores on the three dimensions of mental health (dependent variables), Pearson's correlation coefficient (r) was determined, and then three simple linear regression models were fitted. On the other hand, simple logistic regression modeling was used to determine whether SA influenced depression, anxiety, and stress. The odds ratio (OR) and 95% confidence intervals (95% CI) were obtained for the three models. A p-value <0.05 was considered statistically significant.

## Results

### Participants' characteristics

Among the respondents, 82.55% (175) were female, and 17.45% (37) were male. The majority, 80.66% (171), were 18-19 years old, and 96.22% (204) were single. Most of the students live with their parents. Regarding cell phone use, 75.47% (160) use it for academic purposes, 71.23% (151) for social media, and 46.70% (99) for more than 5 hours a day (Table 1).

### Prevalence of anxiety, depression, stress, and SA among students

Among the students, 52.36% (111) presented with depression, 49.06% (104) anxiety, and 39.15% (83) stress. Additionally, 15.57% (33) of respondents had extremely severe anxiety, 7.55% (16) had severe depression, and 16.04% (34) had moderate stress (Table 2). SA was present in 38.20% (81) of the students, of which 6.60% (14) were male, and 31.60% (67) were female.

**Table 1:** Participants’ characteristics.

Variable	n	%
Sex		
Female	175	82.55
Male	37	17.45
Age		
18-19	171	80.66
20-22	32	15.09
≥23	9	4.25
Marital status		
Single	204	96.22
Cohabitation	4	1.89
Married	3	1.42
Divorced	1	0.47
People you currently live with		
Parents	154	72.17
Others	33	16.04
Alone	13	6.13
Partner	8	3.77
Friends	4	1.89
Phone use		
Academic purpose	160	75.47
Social media	151	71.23
Play video games	27	12.74
Listen to music	106	50.00
Talk to family and friends	134	63.21
Hours spent on the phone		
Less than an hour	3	1.41
1 to 3 hours	46	21.70
4 to 5 hours	64	30.19
More than 5 hours	99	46.70

**Table 2:** DASS-21 Anxiety, depression, and stress levels.

Category	Depression (111) % (n)	Anxiety (104) % (n)	Stress (83) % (n)
Mild	17.92 (38)	14.15 (30)	11.79 (25)
Moderate	16.98 (36)	9.91 (21)	16.04 (34)
Severe	7.55 (16)	9.43 (20)	7.55 (16)
Extremely severe	9.91 (21)	15.57 (33)	3.77 (8)

### Association between DASS-21 subscales and smartphone addiction

Among the students, 25.47% (54) had SA and depression, 24.06% (51) had anxiety and SA, and 21.70% (46) reported stress and SA. The chi-squared test showed significant differences in depression, anxiety, and stress between the group with SA and the group without SA (p <0.05). No statistically significant differences were found between SA and sex (p=1.00). Additionally, 35.81% (76) of respondents with SA were 18-19 years old (Table 3).

Depression subscale scores on the DASS-21 ranged from 0 to 21, with a median score of 3 and an interquartile range of6.25. The median depression score in the non-SA group was 4, while the median score in the SA group was 6. As shown in Table 3, the Mann-Whitney U test showed that the median scores of the DASS-21 subscales were higher in the SA group (p < 0.001) (Table 3).

**Table 3:** Association between DASS-21 subscales and scores and SA.

	Total (212) % (n)	No smartphone addiction (131) % (n)	Smartphone addiction (81) % (n)	^p^
Depression*				0.001
No	47.64 (101)	34.91 (74)	12.74 (27)	
Yes	52.36 (111)	26.89 (57)	25.47 (54)	
Anxiety*				0.002
No	50.94 (108)	36.79 (78)	14.15 (30)	
Yes	49.06 (104)	25.00 (53)	24.06 (51)	
Stress*				<0.001
No	60.85 (129)	44.34 (94)	16.51 (35)	
Yes	39.15 (83)	17.45 (37)	21.70 (46)	
Sex*				1.00
Male	17.45 (37)	10.85 (23)	6.60 (14)	
Female	82.55(175)	50.94 (108)	31.60 (67)	
Age**				<0.001
18-19	80.66 (171)	44.81 (95)	35.81 (76)	
20-22	15.09 (32)	13.21 (28)	1.89 (4)	
>23	4.25 (9)	3.77 (8)	0.47 (1)	
Depression M (IQR)a	5.00 (6)	4.00 (6)	6.00 (8)	<0.001
Anxiety M (IQR)a	3.00(6.25)	3.00 (5)	5.00 (7)	<0.001
Stress M (IQR)a	6.00(7.25)	4.00 (6)	8.00 (6)	<0.001

* Chi-squared test; ** Fisher's exact test; M: Median; IQR: Interquartile range (Q3-Q1); a *Mann-Whitney U test; p: p-value.*

### Regression models

According to the results in Table 4, students with SA are more likely to have depression (OR=2.57; 1.46 4.66), anxiety (OR=2.50; 1.42-4.46), and stress (OR=3.34; 1.87-6.02) compared to students without SA.

**Table 4:** Simple logistic regression models for depression, anxiety, and stress with smartphone addiction.

	ORc	95% CI	^p^
Depression Smartphone addiction	2.57	(1.46-4.66)	<0.001
Anxiety Smartphone addiction	2.50	(1.42-4.46)	<0.001
Stress Smartphone addiction	3.34	(1.87-6.02)	<0.001

*OR: Odds ratio; 95% CI: 95% confidence interval; p: p-value.*

Correlations between DASS-21 and SAS-SV subscale scores were positive and statistically significant: depression (r = 0.34; p <0.001), anxiety (r = 0.31; p <0.001), and stress (r = 0.36; p <0.001).

As shown in Table 5, simple linear regression analysis indicated that SAS-SV scores were significantly associated with DASS-21 subscale scores. The interpretation of the simple linear regression model for the case of depression is as follows: if the SAS-SV scores increase by one unit, the scores associated with the depression subscale of the DASS-21 increase by 0.18 units. The interpretation for the anxiety and stress subscales is similar.

**Table 5:** Simple linear regression models between depression, anxiety, and stress scores and SAS-SV scores.

		95% CI	^p^
Depression SAS-SV	0.18	(0.11-0.25)	<0.001
Anxiety SAS-SV	0.16	(0.10-0.22)	<0.001
Stress SAS-SV	0.20	(0.12-0.26)	<0.001

*P: Estimated regression coefficient; 95% CI: 95% Confidence Interval. SAS-SV: Smartphone Addiction Scale-Short Version; p: p-value.*

## Discussion

To our knowledge, this is the first study in Mexico to examine the prevalence of smartphone addiction and its association with depression, anxiety, and stress. One of the highlights of this research is related to the high prevalence of depression, anxiety, and stress, as well as a moderate prevalence of cell phone addiction, with percentages similar to those reported in other studies^7^^, ^^8^. It should be noted that other studies have found a much higher prevalence of smartphone addiction. For example, a study conducted in Brazil^23^ among nursing students reported a prevalence of 62.60%, which is higher than the 38.20% found in our study.

In the present study, simple linear regression models showed a positive relationship between the smartphone addiction score of the SAS-SV and DASS-21 subscale scores. This finding is consistent with that recently reported by Sarhan^7^ who found that DASS-21 scores were positively correlated with subscales of depression (r = 0.375; p <0.01), anxiety (r = 0.253; p <0.01), and stress (r = 0.328; p <0.05). Similarly, another study showed positive correlations between SAS-SV scores and depression (r = 0.474), anxiety (r = 0.499), and stress (r = 0.461) scores^17^.

A systematic review of the existing literature by Bouazza et al. ^9^ indicates that depression, anxiety, and stress have been strongly correlated with smartphone addiction in most studies reviewed.

The results of simple logistic regression analysis showed a significant association between smartphone addiction, depression, anxiety, and stress among students, which is supported by the study conducted by Nikolic et al. ^14^. They found that students with smartphone addiction were 1.75 times more likely to have elevated stress levels, 2.04 times more likely to have symptoms of anxiety, and 2.29 times more likely to have depression^14^. However, it is worth noting that in this study, the association between smartphone addiction and stress was stronger than the association between anxiety and depression. This finding differs from that reported in the systematic review by Bouazza et al. ^9^, where the association between cell phone addiction and depression was stronger. Hashemi et al. ^17^ found no significant association between excessive cell phone use and depression.

Most of the studies have been conducted with university students, so a probable cause of depression could be the stressful environment of universities, as well as the lack of leisure space and time. In this sense, the study was conducted with first-year undergraduate students who, as university adolescents, had recently experienced a major change in their lives: the transition from high school to university. This process can affect mental health due to academic demands, learning difficulties, acquisition of new skills for professional development, self-management of schedules, and adaptation to a new educational environment, among other challenges. As a result, students often seek refuge in smartphone applications such as games and social media^9^^, ^^24^. It has been shown that people who overuse smartphones tend to feel more depressed and isolated without their cell phones and may also experience symptoms of worry, intolerance, lack of control, withdrawal, mood swings, conflict, lying, overuse, and loss of interest^25^.

The present study allowed us to obtain evidence, in a university population, mainly young adults, of symptoms of anxiety, depression, and stress related to smartphone addiction. Likewise, it is necessary that nursing students be evaluated to detect possible mental disorders and guide them in an appropriate treatment plan. The results of this study may contribute to the identification of risk factors to implement institutional, educational interventions with preventive and control approaches in psychic disorders that affect mental health and impact the overall well-being of students, as well as their cognition, behavior, motivation, learning, and emotion regulation. In this sense, psychoemotional disorders such as stress, anxiety, and depression in an educational environment manifest themselves in students through symptoms such as excessive preoccupation with academic activities, irritability, decreased ability to concentrate, headaches, difficulty memorizing, hopelessness, low self-esteem, difficulty solving problems, and fatigue. These disorders contribute to poor self-care.

There is a need to explore the impact of smartphone use among university students, regardless of their field of study. In addition, based on the findings on the consequences of the excessive use of smartphones, the integration of a multidisciplinary group for the prevention and management of smartphone addiction is necessary. Nursing plays an important role in health promotion and stands out as one of the main advocates for health education. Through a dynamic and continuous teaching-educational process, this discipline can help this population to improve their knowledge and behaviors, promoting optimal psychophysiological development through humane, in-person, and effective psycho-educational interventions, either in groups or individually. These interventions should enable individuals to understand their problems and address them with more appropriate actions, including stress management techniques, self-esteem development, and physical and sports activities.

During the educational intervention, nursing should be the strongest point of contact, focusing on the learners' needs and problems through the use of discipline-specific knowledge so that students develop attitudes, skills, and self-care techniques aimed at protecting, supporting, and maintaining their emotional well-being. In addition, a possible recommendation would be to increase the level of knowledge among students and teachers about the benefits of smartphones for education while balancing this with information about the health consequences associated with their excessive and unnecessary use.

The study has some limitations. The use of mental health assessment tools, such as the DASS-21, facilitates the identification of cases or probable cases of anxiety, depression, and stress that are clinically useful; however, at no time should they replace the assessment of an expert in the clinical area. In addition, the cross-sectional design of the study, which examined only one point in time, may have limited the generalizability of the research. Well-designed longitudinal and clinical research needed to study this association further. We agree with the findings of several studies recommending that future research should include covariates related to smartphone use and lifestyle, such as physical activity.

## Conclusion

Among nursing students, there was a high prevalence of depression, anxiety, and stress; smartphone addiction was significantly associated with these conditions. It is recommended that the sample size be increased and other possible related factors be identified in future research work in our country. In addition, due to the paucity of literature on the problems associated with this issue in our country, it is suggested that more research be conducted in other age groups, such as adolescents in high school, middle school, and older adults.
